# Conometric Connection for Implant-Supported Crowns: A Prospective Clinical Cohort Study

**DOI:** 10.3390/jcm12247647

**Published:** 2023-12-13

**Authors:** Saturnino Marco Lupi, Dario De Martis, Claudia Todaro, Gaetano Isola, Mario Beretta, Ruggero Rodriguez y Baena

**Affiliations:** 1 Department of Clinical Surgical, Diagnostic and Pediatric Sciences, School of Dentistry, University of Pavia, 27100 Pavia, Italy; saturninomarco.lupi@unipv.it (S.M.L.); dariodemartis@gmail.com (D.D.M.); ruggero.rodriguez@unipv.it (R.R.y.B.); 2 Department of General Surgery and Surgical-Medical Specialties, School of Dentistry, University of Catania, 95124 Catania, Italy; gaetano.isola@unict.it; 3 Department of Biomedical, Surgical and Dental Sciences, University of Milan, 20122 Milan, Italy; mario.beretta@unimi.it

**Keywords:** conometric connection, dental implants, fixed dental prosthesis, clinical outcome

## Abstract

Background: Traditional screw or cemented connections in dental implants present limitations, prompting the exploration of alternative methods. This study assesses the clinical outcomes of single crowns and fixed partial prostheses supported by conometric connections after one year of follow-up. Methods: Twenty-two patients received 70 implants, supporting 33 rehabilitations. Biological responses and prosthodontic complications were evaluated at baseline, 6 months, and 12 months. Results: All implants exhibited successful osseointegration, with no losses or peri-implant inflammation. Marginal bone levels showed minimal changes, well below pathological thresholds. The difference in marginal bone loss (MBL) was −0.27 ± 0.79 mm between T0 and T1, and −0.51 ± 0.93 mm between T0 and T2. No abutment screw loosening or crown chipping occurred. However, coupling stability loss was observed in nine cases. Conclusions: The conometric connection demonstrated successful integration and minimal complications after one year. This alternative shows promise, particularly in simplifying handling and improving marginal adaptation. Further research with larger sample sizes and longer follow-up is warranted for comprehensive validation.

## 1. Introduction

Since the advent of modern implantology in the 1960s, credited to Brånemark [1], the observation of marginal bone resorption has prompted the exploration of connections that mitigate this issue. Various connection types have been proposed, including antirotational, internal or external hexagon, conometric, flat-to-flat, platform-matched, and platform-switching connections. Additionally, suggestions include implants with connections distant from the bone margin or without connection (one-piece implants) [2,3,4,5,6,7]. From the introduction of dental implants, research has aimed to optimize both biological response and mechanical properties [8,9,10]. The stability of soft and hard tissues emerges as a crucial consideration regarding the biological implications of dental rehabilitations. Simultaneously, achieving complete success in rehabilitation necessitates addressing potential mechanical complications [11].

Traditionally, the connection between dental implants and prosthetic superstructures can be either screw-retained or cemented. However, both methods present inherent limitations from both biological and mechanical perspectives [12].

Despite the advantages of cemented restorations, challenges arise due to the need for complete removal of excess cement in the soft tissues surrounding the implant [13]. Studies have demonstrated a strong correlation between residual cement and the onset of chronic peri-implant pathologies [14], emphasizing the complications linked to excess residual cement [15,16]. In vitro testing revealed the presence of cement residues in all tested samples, positioned more apically than the margin of the prosthetic crown [17,18]. From a clinical standpoint, it has been verified that an excess of cement can lead to peri-implantitis [14]. However, the precise role of cement in the etiology of peri-implant pathologies remains unclear. The literature suggests that cement remnants may act as an irritant for soft tissues, drawing an analogy with tartar in teeth implicated in periodontal disease [19]. Additionally, bacterial overinfection or a potential toxic reaction to soft tissue cement may occur [17,20].

Research involving experimentally induced peri-implant mucositis in humans has identified bacterial plaque as the etiological agent for peri-implant diseases [18], highlighting the restoration of peri-implant tissue health upon plaque removal [21].

Biological complications may arise from excess cement in the peri-implant sulcus or an incorrect prosthetic margin. Mechanical complications associated with screw retention include the presence of an access hole for the screw or the inability to access the screw itself [22,23,24,25,26]. Screw-type connection allows for ease of removing the superstructure, making it practical for maintenance and repair when facing mechanical complications. Nevertheless, the manufacturing process requires precision, as distortion of the impression material and deformation of the model must be addressed through soldering the superstructure to achieve a passive fit. With screw-retained superstructures, the risk of peri-implantitis or peri-implant mucositis due to residual cement is eliminated. Conversely, inadequate connection between the abutment and superstructure in screw-retained prostheses might directly elevate stress on the mandible. Predictably, such a scenario can have implications for the prognosis of the implants [27].

Natural teeth have a mobility allowance of several dozen micrometers due to the pressure deviation within the periodontal ligament. In contrast, in the case of implant-supported fixed prosthetic dentures, the range of movement is documented to be approximately 10 μm or less [28]. When a slight misfit occurs in the implant superstructure, it is believed that the surrounding tissue experiences greater stress compared to natural teeth, leading to subsequent complications. Studies have shown that a misfit resulting in a microgap at the implant–abutment interface can trigger micromotion and subsequent bone resorption in the surrounding area [29,30]. Additionally, instances of complications, such as abutment screw loosening and diminished preload, have been reported when misfit exists between abutments and superstructures, resulting in fractures of superstructures and components [31,32,33]. Minimal biological and mechanical complications have been reported when there is no misfit between the implant and the abutment, and a good passive fit is achieved [34,35].

It is well known that the good health of soft tissues is related to and is an essential condition for the health of hard tissues and marginal bone level [36]. Untreated mucositis conditions could evolve into peri-implantitis, which in turn leads to permanent damage that can result in implant loss [37,38,39].

Therefore, in an attempt to improve traditional retention methods, the conometric connection was introduced. The conometric connection relies on the frictional effect between the abutment and coping. A perfect fit between the abutment and coping leads to good marginal adaptation with a biological seal of the inner mechanical compartment. The perfect adaptation between the two components is achieved through the use of industrially manufactured materials [40]. The absence of cementation and presence of a perfect fit between the components therefore eliminates two of the peri-implantitis etiological factors. Prosthetic retention is guaranteed, as previously mentioned, thanks to a perfect fit, but above all, thanks to the physical phenomenon of friction between bodies [41]: this force characterizes two bodies intimately in contact and is opposed to the reciprocal motion. The retentive force decreases as the angle between the cone and the perpendicular to the base increases. In other words, a larger angle results in a smaller retentive force. Conversely, if the walls tend towards parallelism, the retention force will be at its maximum [42]. Retention for structures that include more than one implant abutment, in addition to conicity, is also influenced by the degree of parallelism of abutments [41]. A disparity of parallelism up to 5 + 5 degrees is tolerated without affecting retention [43]. Through 5000 insertion–separation cycles in vitro, it was shown that the retentive force remains nearly constant. Thus, the conometric connection system was observed to generate a consistent and appropriate retentive force over an extended period [44]. 

The prefabricated industrial nature of this connection makes it highly suitable for a digital approach. A digital workflow could potentially involve scanning the spatial position of the abutment once the precise conformation of the abutment is determined [45]. The technological progress in computer-guided implant planning, utilizing digital scanning and 3D radiology, enhances predictability in digitally determining the angle of implant abutments and facilitates its translation into the surgical domain. In guided surgery, the design software enables the planning of both implant positions and abutment angles, leading to the attainment of optimal parallelism [43]. The intersection of conometry and comprehensive digital CAD/CAM is intriguing. Indeed, potential occlusal or marginal discrepancies arising from scanner or software approximation errors might be circumvented during the secondary cementation process. The subgingival placement of the prosthetic margin does not involve an inflammatory risk due to an excess of cement or a gap [40]. SEM analysis of the interface zones between coping and abutment, after the system has been subjected to a load, did not show an appreciable gap; therefore, this entails a lower risk of bacterial colonization [46]. Also, in vitro studies showed that biological sealing avoids bacterial contamination [45]. 

This type of connection could be very easy to handle for the insertion–removal procedure and for the management of marginal adaptation with no gap or cement excess [47]. Furthermore, professional hygiene procedures could be less time consuming due to this characteristic [48]. The conometric connection seems to demonstrate good retention force in comparison with traditional ones [41,49]. A limitation lies in the necessity for the conometric connection to be well positioned and fully fitted to achieve the appropriate level of retention [47].

In the literature, this type of connection has been scarcely studied, and there are even fewer prospective clinical studies in humans [45].

The aim of the present study was to evaluate the clinical outcome of single crowns and fixed dental prostheses (FDPs) supported by conometric connections and dental implants after one year of follow-up.

## 2. Materials and Methods

This clinical prospective observational study received approval from the I.R.C.C.S. San Matteo, Pavia Ethical Commission (approval no. 0057089/22) and was conducted in accordance with the recommendations of good clinical practice. The study did not receive any financial grants, but it obtained, free of charge, the prosthetic material used in the study from Luigi Ornaghi S.N.C (Brugherio, Italy). No researcher who participated in the study received funds from the company.

### 2.1. Patient Selection

During the period from December 2021 to May 2023, participants for this study were recruited from the pool of patients at the Department of Oral Surgery and Implantology, School of Dentistry, University of Pavia, Pavia, Italy, who required implant-prosthetic fixed rehabilitations. After providing a detailed explanation of the study protocol, the patients who chose to participate in the study signed an informed consent form and were subsequently enrolled. All patients included in this study were over 18 years of age and were in good health (ASA1–2) [50,51].

To be included in the study, all patients must have fulfilled the following inclusion criteria:

(i) ≥18 years old;
(ii) Type 4 (completely healed sites) according to Hammerle’s classification [52];
(iii) Compliance with good oral hygiene;
(iv) Had a favorable and stable occlusal pattern with dentitions in the opposite side.

The following exclusion criteria were applied:

(i) Patients with immuno-compromised status;
(ii) Uncontrolled diabetes mellitus;
(iii) Current malignancies;
(iv) History of radiation therapy in the head and neck region;
(v) Chemotherapy within 5 years prior to surgery;
(vi) Current treatment with steroids and/or bone antiresorptive agents;
(vii) Neurological or psychiatric handicap that could interfere with good oral hygiene;
(viii) Present drug and/or alcohol abuse;
(ix) Inadequate compliance.

Smoking was not regarded as an exclusion criterion.

### 2.2. Surgical Protocol

The surgical protocol for implant placement used in this study was the one usually employed in everyday clinical practice [53]. 

All patients received endosseous dental implants (Ornaghi Conical Grade Implant^®^, Luigi Ornaghi S.N.C, Brugherio, Italy), using either a one- or two-stage approach. This type of implant features an internal hexagon connection with a double-cone design, having a 4° taper at the apex. The conical closure is sized to allow the application of the platform-switching concept [54]. In the coronal area of the implant, the cortical collar grooves are dimensioned to ensure an appropriate distribution of masticatory loads. The cortical grooves are located along the tapered portion of the implant collar and have a rounded morphology. This type of implant is manufactured with three macro-grooves at the apical portion. During device insertion, the cutting macro-grooves collect bone portions, preserving them. This type of implant features a newly designed spiral (Nest-Shape^®^). The main features of the new spiral morphology include both relevant biological and biomechanical functions. The spiral geometry is crucial in the bone healing phase: the double 25-micron concavities on the entire spiral and implant body significantly extend the contact surface with the bone, influencing the primary distribution of newly formed bone. In fact, the initial osteoblasts, vessels, and bone trabeculae primarily concentrate in the spiral concavities, and only after 30 days from implant insertion can a homogeneous distribution on the implant surface be observed. Osteoblastic proliferation is confirmed by increased alkaline phosphatase formation and the presence of PGE2 and TGF-beta [55]. Besides benefiting the healing phase, the concavities allow a substantial reduction in the bone cutting section, making the spiral atraumatic for the implant alveolus, despite its 0.5 mm height. This new spiral morphology has a profile with a constant pitch along the entire length of the implant. Specifically, the pitch, measuring 0.9 mm, makes the device versatile and significantly reduces insertion time. This spiral has the peculiarity of having three grooves within its profile. These grooves, with a radius of 0.25 mm, allow a significant reduction in the cutting section of the spiral and greatly increase the surface area for osseointegration. Another important feature of the new spiral morphology is that during implant insertion into the implant alveolus, the spiral mechanically engages with the medullary bone. The implant site, which must be prepared according to protocol with specific drills, allows blood to flow and distribute over the entire implant body, stimulating rapid formation of young and well-oxygenated blood clots. This new spiral morphology maintains a constant depth along the entire length of the implant, except in the coronal zone, where it reduces. The 0.5 mm depth of the spiral gives the profile an average value of implant alveolus invasion. The diameters used were 3.75 and 4.2 mm, with implant lengths ranging between 8 and 12 mm. Twelve weeks after surgery, osseointegration was assessed clinically and radiographically; subsequently, implants were uncovered if necessary, and the prosthetic procedures started. All implants were placed by a single surgeon with more than 40 years of experience in the field of oral implants.

### 2.3. Prosthetic Protocol

The components of this system involve the use of a transmucosal abutment (Ornaghi Prasa^®^, Luigi Ornaghi S.N.C, Brugherio, Italy) directly connected to the implant, with a torque of 20 N/cm to be used in place of the healing abutment. The height of the transmucosal abutment can be chosen to adapt to the thickness of the mucosa, ranging from a minimum of 1 mm to a maximum of 4 mm.

**Figure 1:**
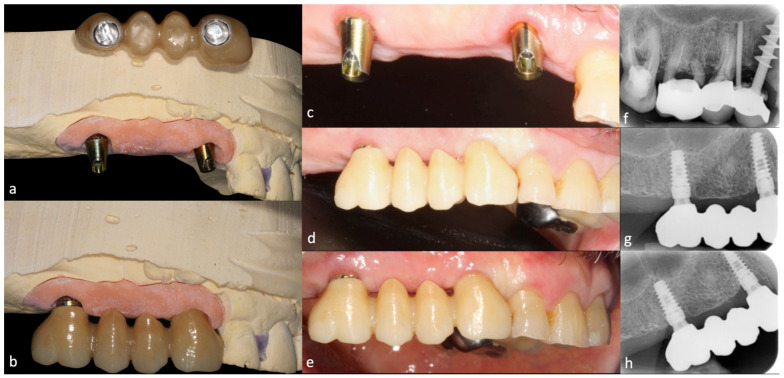
Cast models (**a**,**b**). Clinical images of a representative case at baseline (**c**,**d**) and after 12 months (**e**). Initial periapical radiographs of the initial situation (**f**), after six months (**g**), and after twelve months (**h**).

Conometric abutment is connected to the transmucosal abutment through a screw; the conometric abutment allows for correction of any misalignments up to 24 degrees. A conometric coping, to be cemented in the fixed prosthesis produced with traditional or CAD/CAM techniques, is coupled to the conometric abutment. The precise coupling of the conometric abutment and coping is the determining factor in this retention system (Figure 1).

The implant impression technique in this system can be either direct or indirect. The indirect technique involves taking an impression using either snap-on or screw-retained transfers directly connected to the implant. The transmucosal abutment is placed on the developed model, followed by the remaining prosthetic processing. The direct impression technique involves connecting the transmucosal abutment to the implant and then taking precise impressions. This technique has the advantage of not having to remove and reposition the transmucosal abutment, with positive effects both biologically and in terms of process accuracy. In this study, all precise impressions were made using the direct polyether technique (Impregum™ Penta™, 3M, Saint Paul, MN, USA) with the closed-tray technique.

After preparing the gypsum models with replicas, an appropriate conometric abutment (Ornaghi Prasa^®^, Luigi Ornaghi S.N.C, Brugherio, Italy) was mounted, and the conometric coping was placed on it. Then, the models were scanned, and the crowns were produced in zirconia/ceramic using the CAD/CAM technique commonly employed. The copings were cemented to the crowns with self-polymerizing resin cement (G-CEM ONE™, GC Co., Ltd., Tokyo, Japan). All crowns were fabricated by the same dental technician. The clinical crown delivery procedure was simplified with this componentry and involved connecting the conometric abutment with a screw and placing the prosthetic restoration under pressure. Subsequently, static and dynamic occlusal checks were performed, along with any necessary occlusal adjustments. At the end of the prosthetic procedure, a periapical X-ray was taken to evaluate the accuracy of the rehabilitation, and periodontal indices were recorded.

### 2.4. Follow-Up

Regular follow-up visits were conducted at 6 and 12 months after prosthetic delivery. During these visits, prosthetic complications were registered, and marginal bone level (MBL) was evaluated through periapical X-rays. Due to the unique configuration of the transmucosal abutment, featuring a concave nitrided surface to allow for maximum biological sealing of the peri-implant gingiva, probing of the peri-implant sulcus was only performed in case of inflammation indicators (redness, swelling, gingival recession) detection [56]. In such cases, periodontal indices were recorded, and probing was conducted to determine the bleeding on probing (BOP), measured at four points (mesial, buccal, distal, and lingual) around the implant, yielding a dichotomous result. To reduce the risk of bias, the X-rays were obtained using a Rinn holder by a single operator well trained in the experimental protocol and in the measurement procedures; clinical examinations were conducted by a single operator well trained in the experimental protocol and with twenty years of clinical experience. Maintenance oral hygiene procedures were performed as needed during the follow-up sessions.

### 2.5. Outcomes

All RX images were analyzed using ImageJ (v. 1.53a, NIH, Bethesda, MD, USA) software, and all data were stored and analyzed using Excel^®^ (v. 16.72, Microsoft, Redmond, WA, USA). 

To assess peri-implant inflammation, an initial visual inspection was performed. Another parameter for peri-implant inflammation was the stability of the peri-implant bone, assessed by measuring the MBL on periapical radiographs taken during routine follow-up visits. The average of the two values was then calculated for the analysis of peri-implant bone resorption using this method. For each implant site at the same follow-up moment, MBL data were averaged and stored. The baseline visit and the two follow-up assessments provide three average MBL indexes; for all data considered, standard deviation was calculated.

### 2.6. Statistical Analysis

A sample size of 66 implants was calculated, considering a 5% alpha error, 95% power, a standard deviation of 0.8 mm [57], and a clinically significant minimum difference of 0.5 mm (which is half of the periodontal probe scale). Finally, the sample size was set at 70 implants to account for potential dropouts from the study. Data and averages were analyzed using the F-test and Student’s *t*-test, with a significance level set at 0.05.

## 3. Results

In total, 22 patients were enrolled, consisting of 13 males and 9 females, with a total of 70 implants placed. The patients had an average age of 47 ± 13 years. On average, 2.5 ± 1.3 implants were inserted per patient. The implants supported a total of 33 rehabilitations, including 7 single crowns and 26 fixed partial prostheses. Specifically, 24 were supported by only two implants, and 2 were supported by more than two implants. In total, 76 dental units were positioned (mean = 3.45 per patient). Twenty-seven prostheses were in the posterior position, and six rehabilitations were in the anterior portions.

### 3.1. Biological Response

**Figure 2:**
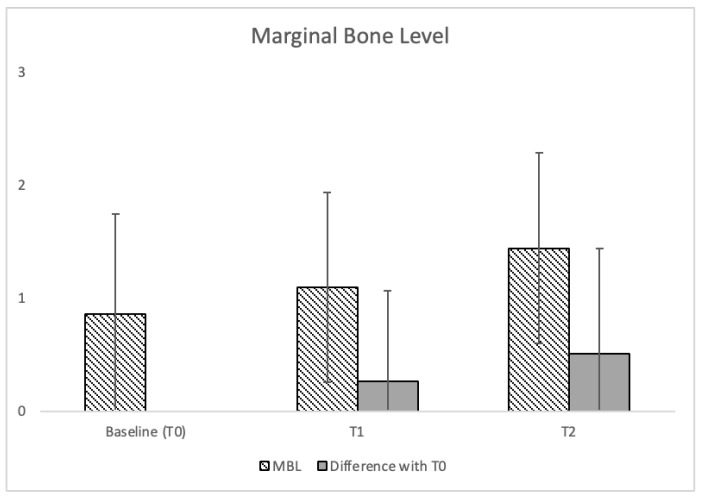
Mean MBL at T0, T1, T2, with standard deviation. Data in millimeters.

All implants exhibited clinical and radiographic features of osseointegration, and no implant was lost at each follow-up. The success rate was 100% at every follow-up interval. In no case was peri-implant inflammation detected (0%) in all follow-up intervals. The average MBL was 0.86 ± 0.89 mm at baseline, 1.10 ± 0.84 mm at T1, and 1.44 ± 0.84 mm at T2. The difference in MBL was −0.27 ± 0.79 mm between T0 and T1, and −0.51 ± 0.93 mm between T0 and T2. MBL at T0 and T2 showed significantly different results (*p* < 0.001) (Figure 2).

### 3.2. Prosthodontic Complications

During the follow-up period, no complications related to abutment screw loosening were observed in any of the 64 implants, nor were there any instances of crown chipping among the 76 dental units. In nine cases, stability losses were recorded in the coupling between the conometric abutment and crown coping. The locations of this group of rehabilitations are summarized in Table 1. 

In case of loss of retention, detached prostheses were cemented with provisional cement (Temp Bond, KERRHAWE S.A., Bioggio, Switzerland); following this procedure, no further detachments were recorded. No further technical complications were observed.

## 4. Discussion

Traditionally, the connection between the abutment and crown is either screwed or cemented [58,59,60]. Both solutions have their advantages and disadvantages. Cemented prostheses are more susceptible to biological complications due to the presence of cement in the peri-implant sulcus. On the other hand, the screwed connection presents issues related to the position of the screw hole, namely aesthetic and occlusal problems. In addition, screw-retained connections are highly susceptible to errors during the impression, potentially leading to misfits that subsequently lead to both biological and mechanical complications. To address this issue, other types of connections have also been proposed, such as the conometric connection [45]. The advantages of the conometric connection include the absence of cement and the absence of a screw access hole, providing aesthetic and occlusal stability benefits. However, there is limited clinical information available on this type of connection. 

A case report aims to propose a technique to improve the accuracy of guided–welded approach planning for immediate restorations supported by conometric abutments [41].

An in vitro study found that the retention strength of this type is influenced by the shape of the abutments and can match or even surpass the retentive strength of commonly used provisional cements [49].

A prospective study conducted on 39 patients with provisional partial rehabilitations using conic coupling retention supported by immediate implants did not observe any complications during a follow-up period of up to 3 years [47]. This study concluded that this type of rehabilitation represented a successful, cost-effective treatment modality. Another prospective clinical study analyzed the treatment outcomes with conometric connections in a sample of 100 implants. At two years, a variation in MBL of 0.4 mm was observed, and no prosthetic detachments were recorded [61]. In another prospective clinical study involving 65 patients and 130 implants, no detachments were observed over a two-year follow-up period, although other prosthetic complications were noted [62]. In this study, the difference in MBL from T0 to T1 was −0.27 ± 0.79 mm, and from T0 to T2, it was −0.51 ± 0.93 mm; these data are at least consistent with what is found in the literature. Furthermore, the loss of MBL was well below the threshold value of 3 mm, considered indicative of peri-implant pathology onset [63]. Furthermore, a bone loss of 1 mm in the first year followed by 0.2 mm per year is considered acceptable [64].

However, detachments were recorded in 27% of the prostheses, a relatively high rate, especially when compared to those reported in the literature [40,47,49]. Since this com-plication was addressed simply by cementing with provisional cement and was no longer observed, it can be considered of minor significance. However, adequate retention is one of the highest expectations of patients, so rehabilitations that do not meet this parameter should be considered carefully [65].

A great possibility offered by the conometric connection, evaluated in the present study, consists in the easy management of the abutment’s height in case of reduced occlusal space.

One of the advantages of this type of connection is the ability to harness the potential of CAD/CAM technologies in new directions. In fact, the prosthetic margin may not be perfectly captured in the impression and may not be perfectly replicated in the crown, as both are mass-produced with extremely high precision characteristics at an industrial level [66]. 

The limitations of this study are represented by the limited follow-up, the small number of treated cases, and the absence of a control group. However, it must be considered that this type of implant-prosthetic rehabilitation has only limited information in the literature, and therefore, a study on this topic is exploratory in nature, serving as a foundation for further research. Therefore, long-term studies with a larger number of cases treated and control groups are necessary to validate the results presented in this study.

## 5. Conclusions

The results of this study suggest the feasibility of a clinical alternative to screw-retained or cemented connections, namely the conometric connection. The findings provide valuable data for the clinical application of this connection, showing that cases treated with this type of connection exhibited excellent biological integration and a stable level of peri-implant tissues up to one year of follow-up. No complications related to screw loosening or ceramic chipping were observed. From the results of this study, it emerges that a potential complication associated with this type of connection could be the disconnection between the abutment and coping. However, this complication was addressed by cementing with provisional cement, and no further technical complications were observed. The conometric connection method allows for high integration with CAD/CAM production systems, showcasing the potential for harnessing technology in new directions. The results of this study need to be confirmed by clinical studies with larger sample sizes and longer follow-up periods to validate the presented findings and assess the long-term success and stability of the conometric connection in implant-prosthetic rehabilitations.

## Figures and Tables

**Table 1:** Data on detachment position.

	Anterior	Posterior
Maxillary	4	2
Mandibular	1	2

## Data Availability

The data presented in this study are available on request from the corresponding author. The data are not publicly available due to privacy reason.
